# Effects of Vonoprazan on Isolated Rat Lower Esophageal Sphincter and Ileum

**DOI:** 10.3390/ph19081199

**Published:** 2026-07-31

**Authors:** Mert C. Ongun, Muhammed Yayla, Nihal Cetin, Hulagu Bariskaner

**Affiliations:** Department of Medical Pharmacology, Faculty of Medicine, Selcuk University, Konya 42130, Turkey; muhammed.yayla@selcuk.edu.tr (M.Y.); nihalcetin84@gmail.com (N.C.); hbkaner@hotmail.com (H.B.)

**Keywords:** vonoprazan, lower esophageal sphincter, ileum, relaxation, smooth muscle contractility, isolated organ bath

## Abstract

**Background/Objectives:** Vonoprazan is a potassium-competitive acid blocker that is increasingly being used in the treatment of gastroesophageal reflux disease. Although its gastric acid suppressive effect is well established, its direct effects on gastrointestinal smooth muscle remain unclear. This study aimed to investigate the effects of vonoprazan on the contractility of isolated rat lower esophageal sphincter (LES) and ileum tissues. **Methods:** Contractile responses of isolated rat LES strips and ileum segments were evaluated in organ baths. The direct relaxant effects of cumulative vonoprazan were assessed in tissues precontracted with carbachol. In addition, the effects of vonoprazan on spontaneous ileal contractions and on cumulative carbachol- and serotonin-induced contractions were examined after 1 h of incubation with different concentrations of vonoprazan. **Results:** Vonoprazan produced concentration-dependent relaxation in both ileum and LES tissues (−logEC50: 4.86 ± 0.16 and 4.08 ± 0.49, respectively). In isolated rat ileum, vonoprazan significantly reduced the normalized amplitude of spontaneous contractions at 100 µM compared with 1 µM (1 µM: 105.40 ± 18.6% vs. 100 µM: 54.84 ± 18.52%, *p* = 0.002). Following 1 h incubation, 10 and 100 µM concentrations of vonoprazan attenuated both carbachol- and serotonin-induced contractions in ileum and LES. **Conclusions:** These results indicate that vonoprazan directly inhibits gastrointestinal smooth muscle contractile activity by decreasing spontaneous ileal contraction amplitudes and attenuating muscarinic and serotonergic contractile responses in isolated rat ileum and LES. Further studies are required to determine the underlying mechanisms and clinical relevance of these effects.

## 1. Introduction

Gastroesophageal reflux disease (GERD) is a disease characterized by the pathological reflux of gastric contents into the esophagus. GERD affects approximately 10–20% of adults worldwide [1]. If left untreated, the disease may lead to complications such as esophagitis, stricture formation, and Barrett’s esophagus, and in the long term, it increases the risk of adenocarcinoma [2]. Pharmacological management of GERD primarily includes proton pump inhibitors (PPIs), potassium-competitive acid blockers (P-CABs), histamine type-2 receptor antagonists, and mucosal protective agents such as sucralfate and alginates [3].

P-CABs are a more recent class of medications that reduce gastric acid secretion other than classical PPIs. They reversibly suppress acid secretion as a result of their competitive inhibition of the H+/K+ ATPase’s potassium binding site [4]. Compared with classical PPIs, vonoprazan has several advantages including a rapid onset of action, reversible inhibition, stable acid suppression, and reduced susceptibility to *CYP2C19* genotype-related variability [5,6]. These established differences in acid suppressive pharmacology also mean that non-gastric actions reported for classical PPIs cannot be directly extrapolated to vonoprazan. Additionally, these pharmacological properties have contributed to its successful use in the treatment of GERD and in *H. pylori* eradication [7]. Although first introduced and utilized in Japan and other Asian countries, recent FDA approvals for erosive GERD in 2023 and non-erosive GERD-related heartburn in 2024 have further emphasized the increasing global role of vonoprazan in GERD management [7,8].

Lower esophageal sphincter (LES) impairment is a major component of the pathophysiology of GERD. By establishing a high-pressure barrier between the stomach and esophagus, the LES helps prevent gastroesophageal reflux [9]. A reduction in LES tone plays a crucial role in GERD development [10]. Isolated rat LES preparations have been used in organ bath studies to evaluate the effects of pharmacological agents on sphincter tone [11,12]. Although LES dysfunction is central to GERD pathophysiology, motor function beyond the LES may also influence reflux characteristics. In a pH impedance study, delayed gastric emptying was associated with a greater number of liquid or mixed and postprandial reflux events, prolonged bolus clearance, and greater proximal extension of reflux, although it was not associated with increased esophageal acid exposure [13]. A prospective clinical study demonstrated that vonoprazan administration delayed gastric emptying in healthy adults, suggesting that its potential effects on gastrointestinal motor function may extend beyond the antireflux barrier [14].

The rat ileum exhibits spontaneous rhythmic contractions and measurable agonist-induced responses, making it a suitable ex vivo preparation for evaluating both basal- and receptor-mediated intestinal contractility. Accordingly, isolated rat ileum preparations are widely used to assess the effects of pharmacological agents on spontaneous and agonist-induced smooth muscle contractility [15,16], including the motility-related effects of acid suppressive drugs such as proton pump inhibitors [17,18].

Several isolated organ bath studies suggest that classical PPIs may exert direct effects on smooth muscle preparations independently of acid suppression. In rat LES, PPIs have been reported to relax carbachol-precontracted tissues and to suppress agonist-induced contractile activity [11,12]. Similar inhibitory effects have also been described in rat ileum, vascular smooth muscle, and human myometrium, suggesting that PPIs may influence smooth muscle contractility across different tissues [17,19,20,21]. These findings provide a pharmacological precedent for investigating whether acid suppressive drugs can directly modify smooth muscle function. Nevertheless, because vonoprazan is a P-CAB with a distinct structure and mode of proton pump inhibition, effects on the smooth muscles reported for classical PPIs cannot be assumed to directly apply to vonoprazan.

Current evidence regarding vonoprazan and gastrointestinal motor function is limited. Clinically, vonoprazan has been associated with delayed gastric emptying and constipation; however, these observations do not establish whether vonoprazan directly affects gastrointestinal smooth muscle contractility. Although relaxant effects of classical PPIs have been reported in several smooth muscle preparations, direct acid-independent actions of vonoprazan on isolated gastrointestinal tissues remain unknown. To our knowledge, no previous study has evaluated vonoprazan’s direct effects on LES or ileal contractility. Therefore, the present study investigated the effects of vonoprazan on isolated rat lower esophageal sphincter and ileum tissues.

## 2. Results

### 2.1. Effects of Vonoprazan on Spontaneous Contractile Activity of Isolated Rat Ileum

**Figure 1:**
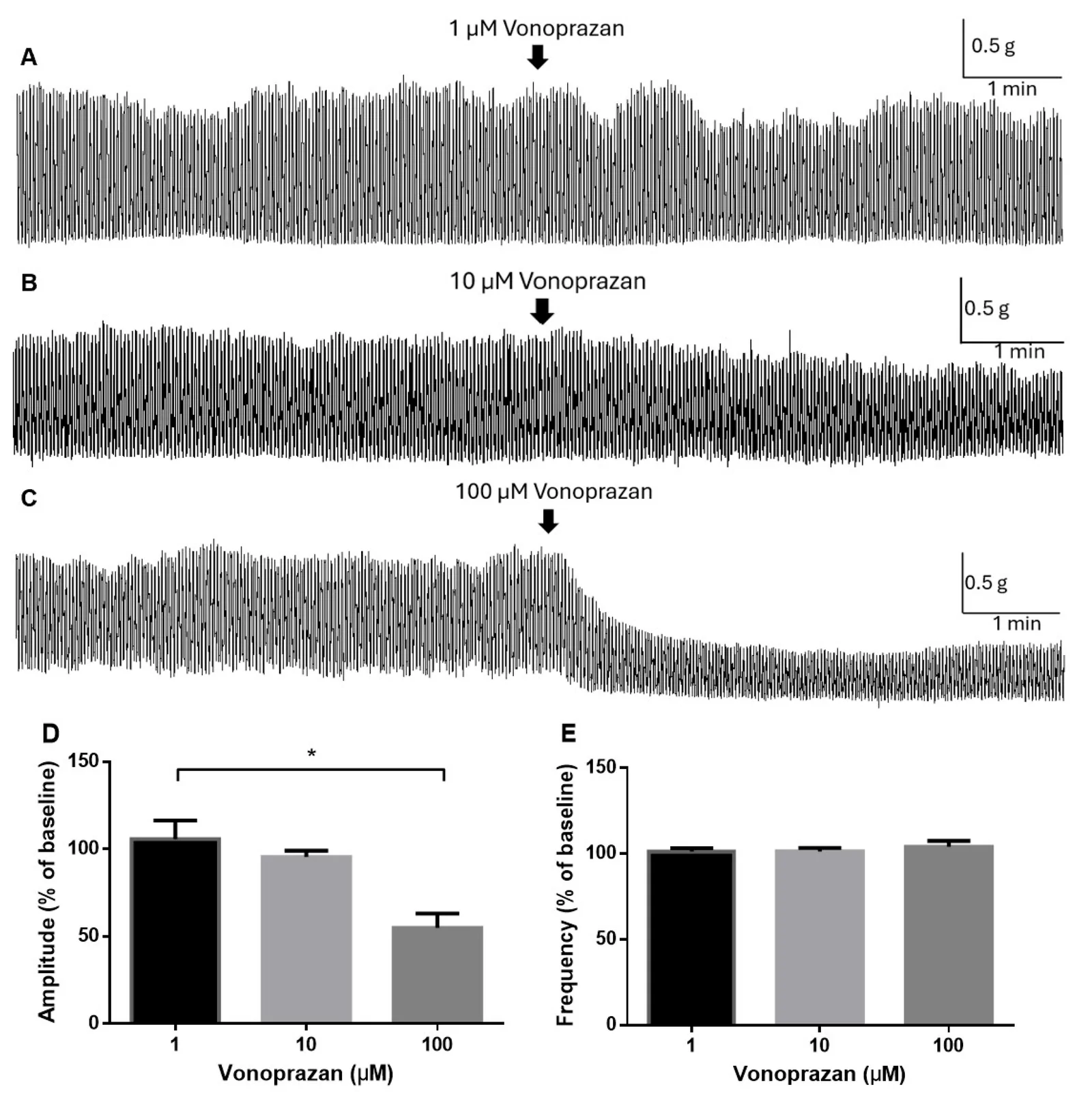
Effects of vonoprazan on spontaneous contractions of isolated rat ileum. Representative original traces show spontaneous ileal contractions before and after the application of 1 µM (**A**), 10 µM (**B**), and 100 µM (**C**) vonoprazan. Arrows indicate the time of vonoprazan administration. Vertical and horizontal scale bars represent 0.5 g and 1 min, respectively. Post-treatment contraction amplitude (**D**) and frequency (**E**) were normalized to the corresponding pretreatment baseline value of each tissue segment and expressed as a percentage of baseline. Data are presented as means ± SEM. Differences among groups were analyzed using one-way ANOVA followed by Tukey’s post-hoc test. (*n* = 5, * *p* = 0.006).

Vonoprazan significantly reduced the baseline normalized amplitude of spontaneous contractions of isolated rat ileum at 100 µM compared with 1 µM (1 µM: 105.40 ± 18.6% vs. 100 µM: 54.84 ± 18.52%, *p* = 0.002; Figure 1A–D). On the other hand, vonoprazan did not significantly affect the baseline normalized frequency of spontaneous contractions (1 µM: 101.20 ± 1.59% vs. 100 µM: 104.10 ± 3.36%, *p* = 0.703; Figure 1E). Pretreatment baseline amplitude and frequency did not differ significantly among the experimental groups (Supplementary Figure S1, *p* > 0.05).

### 2.2. Relaxing Effect of Vonoprazan on Isolated Rat Ileum and LES

**Figure 2:**
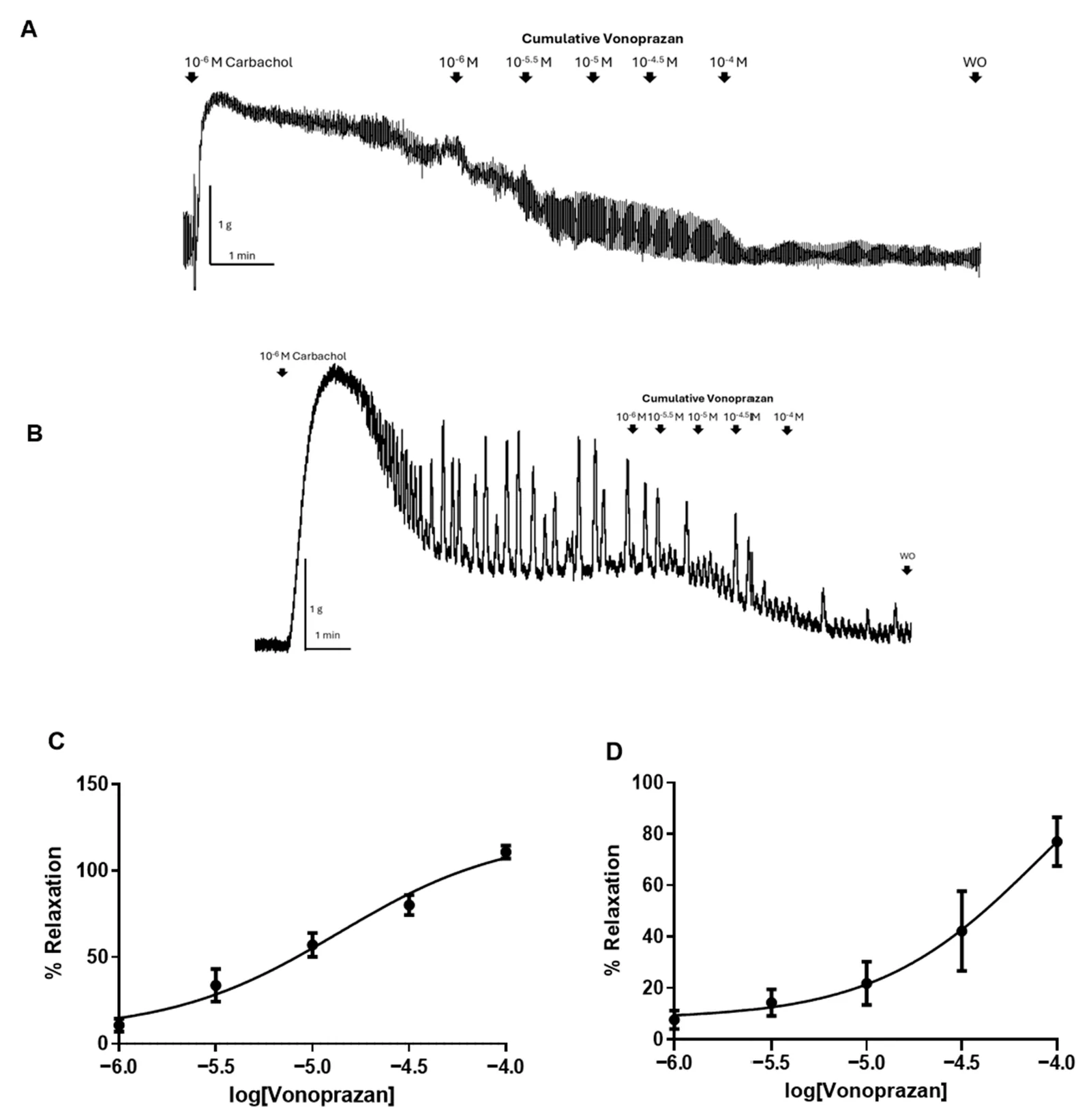
Concentration response curves for vonoprazan induces relaxations on isolated rat ileum ((**A**,**C**), *n* = 6, −logEC_50_: 4.86 ± 0.16) and LES ((**B**,**D**), *n* = 5, −logEC_50_: 4.08 ± 0.49). The −logEC_50_ values did not differ significantly between tissues according to the extra-sum-of-squares F test [F(1,49) = 3.334, *p* = 0.074]. Data are presented as means ± SEM.

The relaxant effect of vonoprazan was examined in ileum segments and LES strips precontracted with carbachol (1 µM). Vonoprazan relaxed ileum segments and LES strips in a concentration-dependent manner (Figure 2A–D). The maximum observed relaxation was 110.00 ± 3.77% in the ileum and 77.05 ± 9.49% in the LES, while the corresponding −logEC_50_ values were 4.86 ± 0.16 and 4.08 ± 0.49 respectively (Figure 2C,D). Comparison of the −logEC_50_ values using the extra-sum-of-squares F test revealed no statistically significant difference between the ileum and LES [F(1,49) = 3.334, *p* = 0.074].

### 2.3. Effect of Vonoprazan on Carbachol- and Serotonin-Induced Contractions in Isolated Rat Ileum

**Figure 3:**
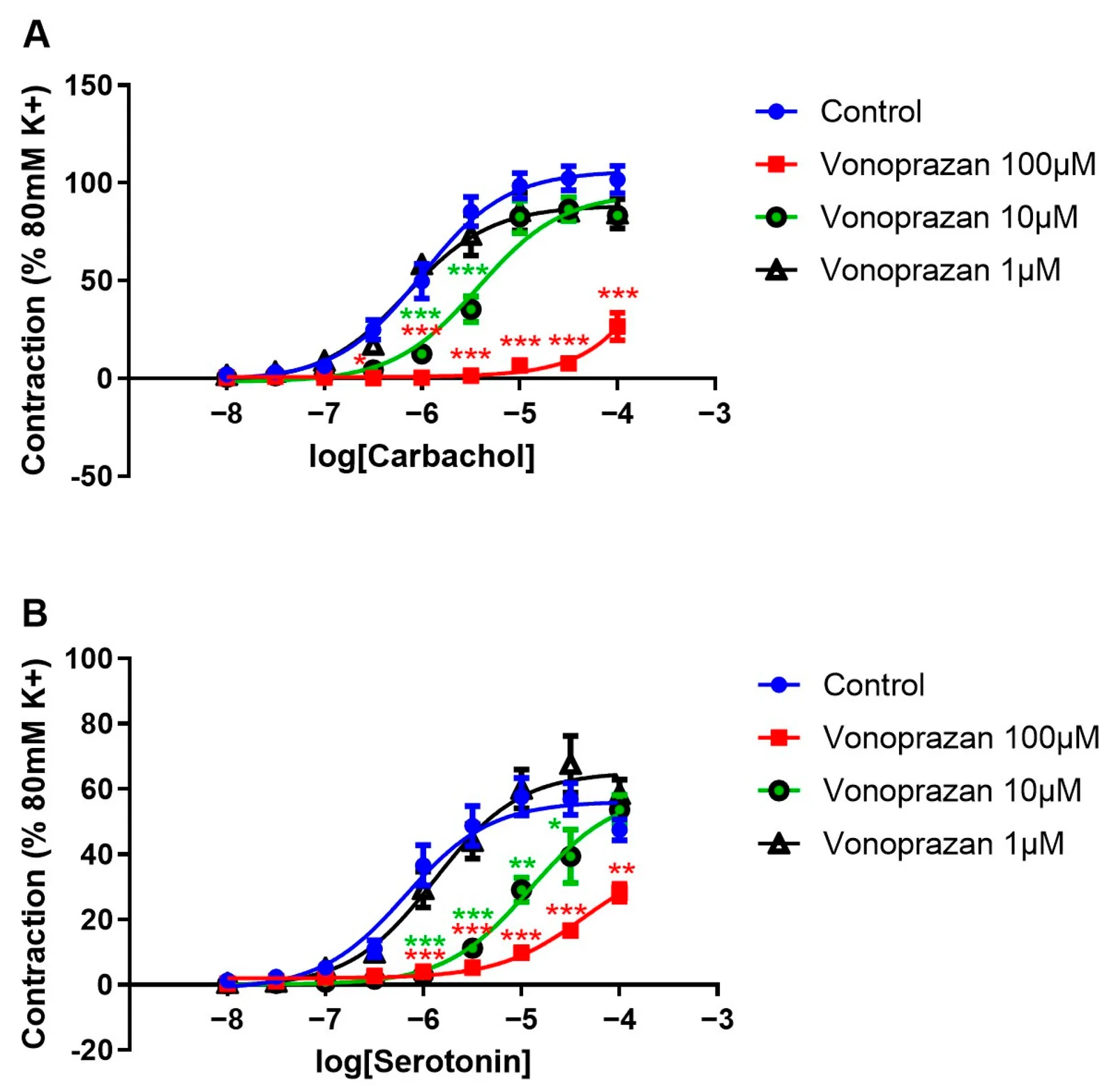
The effects of vonoprazan (1–100 µM) on (**A**) carbachol (*n* = 4)- and (**B**) serotonin (*n* = 4)-induced cumulative concentration response curves in rat isolated ileum. (Repeated measures two-way ANOVA followed by Dunnett’s post-hoc test, * *p* < 0.05, ** *p* < 0.01 *** *p* < 0.001 vs. control). Data are presented as means ± SEM. Adjusted *p*-values for subfigures (**A**,**B**) are provided in Supplementary Tables S1 and S2, respectively. Representative original traces corresponding to subfigures (**A**,**B**) are presented in Supplementary Figures S2 and S3, respectively.

The effects of cumulative administration of carbachol (10^−8^–10^−4^ M) and serotonin (10^−8^–10^−4^ M) were assessed in the absence and presence of 10^−6^, 10^−5^, and 10^−4^ M of vonoprazan. In the rat isolated ileum, carbachol elicited concentration-dependent contractions. Pre-incubation with vonoprazan inhibited these contractions in a concentration-dependent manner. Vonoprazan at 1 µM did not significantly alter the carbachol concentration response curve (Figure 3A, *p* > 0.05). Vonoprazan at 10 µM significantly reduced the contractile response at carbachol concentrations of 10^−6^–10^−5.5^ M (Figure 3A, *p* < 0.001). Vonoprazan at 100 µM significantly reduced the contractile response at carbachol concentrations of 10^−6^ M and higher (Figure 3A, *p* < 0.001).

Similarly, serotonin-induced concentration-dependent contractions in isolated rat ileum. Vonoprazan at 100 µM markedly inhibited serotonin-induced contractions, with significant reductions observed from 10^−6^ M and above serotonin concentrations (Figure 3B, *p* < 0.01–0.001). Incubation with 10 µM of vonoprazan also significantly reduced the contractile response at serotonin concentrations ranging from 10^−6^ to 10^−4.5^ M (Figure 3B, *p* < 0.05–0.001).

### 2.4. Effect of Vonoprazan on Carbachol- and Serotonin-Induced Contractions in Isolated Rat LES

**Figure 4:**
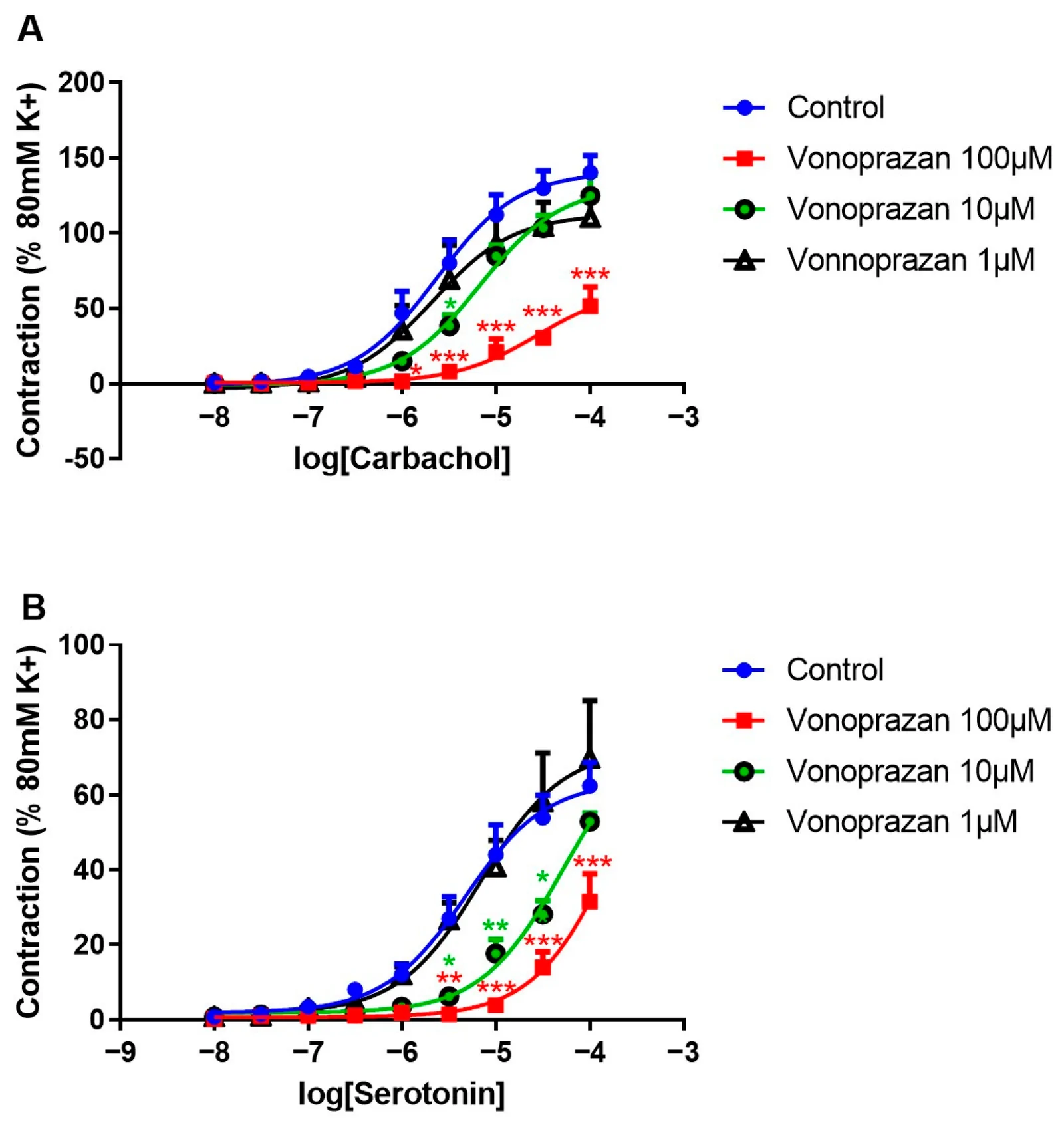
The effects of vonoprazan (1–100 µM) on (**A**) carbachol (*n* = 4)- and (**B**) serotonin (*n* = 4)-induced cumulative concentration response curves in rat LES. (Repeated measures two-way ANOVA followed by Dunnett’s post-hoc test, * *p* < 0.05, ** *p* < 0.01 *** *p* < 0.001 vs. control). Data are presented as means ± SEM. Adjusted *p*-values for subfigures (**A**,**B**) are provided in Supplementary Tables S3 and S4, respectively. Representative original traces corresponding to subfigures (**A**,**B**) are presented in Supplementary Figures S4 and S5, respectively.

The effects of cumulative administration of carbachol (10^−8^–10^−4^ M) and serotonin (10^−8^–10^−4^ M) were assessed in the absence and presence of 10^−6^, 10^−5^, and 10^−4^ M of vonoprazan in isolated rat LES. Vonoprazan at 1 µM did not significantly alter the carbachol concentration response curve (Figure 4A, *p* > 0.05). Vonoprazan at 10 µM concentration significantly reduced the contractile response at a carbachol concentration of 10^−5.5^ M (Figure 4A, *p* < 0.05). Vonoprazan at 100 µM significantly reduced the contractile response at carbachol concentrations of 10^−6^ M and higher (Figure 4A, *p* < 0.05–0.001).

Similarly, vonoprazan at 100 µM markedly inhibited serotonin-induced contractions, with significant reductions observed from 10^−5.5^ M of serotonin and higher concentrations (Figure 4B, *p* < 0.01–0.001). The 10 µM of vonoprazan incubation also significantly reduced the contraction response at serotonin concentrations ranging from 10^−5.5^ to 10^−4.5^ M (Figure 4B, *p* < 0.05–0.01).

## 3. Discussion

The findings of this study demonstrate that vonoprazan elicits concentration-dependent relaxation on isolated rat ileum and LES. Vonoprazan also reduced the amplitude of spontaneous ileal contractions without affecting contraction frequency. Furthermore, pre-incubation with vonoprazan attenuated carbachol- and serotonin-induced contractions. The contractile responses to cumulative carbachol and serotonin in both ileum and LES were significantly attenuated in the presence of vonoprazan in a concentration-dependent manner. To our knowledge, this is the first study to demonstrate that vonoprazan induces relaxation in isolated rat LES and ileum and reduces the amplitude of spontaneous ileal contractions.

Initially, we examined the effects of vonoprazan on spontaneous ileal contractions. Vonoprazan statistically significantly reduced the amplitude of spontaneous contractions at 100 µM, but it did not affect contraction frequency. In an isolated intestine, the timing and frequency of rhythmic activity is largely governed by slow wave generation paced by interstitial cells of Cajal [22]. The amplitude of each contraction reflects how effectively excitation is translated into contraction force. Our results, therefore, suggest that vonoprazan reduces the contractile capacity of ileal smooth muscle without interfering with the underlying rhythmic activity.

Only a few studies have examined the effects of classical PPIs on spontaneous intestinal contractions. In one study, rats received omeprazole twice daily for four days, and the omeprazole suppressed spontaneous activity in isolated ileal segments and reduced intestinal transit [17]. In the study by Kurt et al. (2011), incubation of omeprazole, pantoprazole, and lansoprazole significantly reduced both the amplitude and frequency of spontaneous ileal contractions over the 10^−6^–10^−4^ M concentration range in isolated rat ileum [18]. Our results showed that vonoprazan significantly reduced only the amplitude of spontaneous contractions at 100 µM. Together, these findings suggest that vonoprazan does not strongly engage interstitial cells of Cajal-dependent pacemaker activity.

We next examined the direct relaxing effect of vonoprazan in isolated rat ileum precontracted with carbachol. Vonoprazan produced concentration-dependent relaxation in isolated ileum segments. While translating in vitro tissue responses directly to clinical outcomes requires caution, these direct smooth muscle effects provide a pharmacological basis for known adverse events. In clinical trials and post-marketing surveillance, constipation has been observed as a side effect of vonoprazan [23,24]. Importantly, a prospective clinical trial reported that administration of 20 mg of vonoprazan for 14 days delayed gastric emptying in healthy adults [14]. Our results may provide a basis for the observed effects by demonstrating that vonoprazan reduces the contractile force of intestinal smooth muscle. We hypothesize that the reduction in contractile force observed in our in vitro model could theoretically contribute to clinical observations such as constipation and delayed gastric emptying. It might also hold significance for GERD pathophysiology, as delayed stomach emptying and gastric distension are known to increase the likelihood of reflux events. Conversely, if such smooth muscle relaxation occurs clinically, it could hypothetically offer secondary benefits in patients with spasm-related abdominal pain.

In addition, we investigated the direct relaxant effect of vonoprazan in isolated rat LES precontracted with carbachol. Vonoprazan produced concentration-dependent relaxation in LES strips (−logEC_50_: 4.08 ± 0.49, Figure 2D). Previous isolated organ bath studies have shown that classical PPIs can directly suppress LES contractility. Pantoprazole was reported to relax carbachol-precontracted rat LES preparations, suggesting that PPIs may reduce LES tone independently of their acid suppressive action [12]. In another study, omeprazole, lansoprazole, and pantoprazole relaxed carbachol-precontracted rat LES strips and suppressed carbachol-, angiotensin-II-, and electrical-field-stimulation-induced contractions. They reported −logEC_50_ values of 3.90 ± 0.13, 4.60 ± 0.21, and 4.90 ± 0.17 for omeprazole, lansoprazole, and pantoprazole, respectively, for relaxation of carbachol-precontracted rat LES strips [11]. Thus, the apparent relaxant potency of vonoprazan was broadly comparable to that of omeprazole but lower than those reported for lansoprazole and pantoprazole. However, this comparison is descriptive because differences in experimental protocols, tissue preparations, and curve-fitting procedures may affect potency estimates across studies. These findings extend previous observations with classical PPIs by demonstrating that vonoprazan, a potassium-competitive acid blocker, can also directly relax rat LES smooth muscle.

Under physiological conditions, the LES prevents the reflux of gastric contents into the esophagus by maintaining pressure higher than that of the stomach. In GERD, the barrier function of the LES is compromised, allowing gastric contents to reflux into the esophagus, which leads to tissue damage [3]. Therefore, a drug that inhibits LES contractions, even while successfully suppressing acid secretion, may adversely affect the disease course by impairing LES function.

In approximately 30% of patients receiving PPI therapy, GERD symptoms persist despite the successful suppression of acid secretion [25]. This finding is consistent with the results of a study using impedance pH monitoring in patients with refractory non-erosive reflux disease (NERD). In that study, positive reflux symptoms were associated with non-acidic reflux in 60% of patients with refractory NERD [26]. Similarly, a retrospective clinical study evaluating vonoprazan refractory GERD reported no cases of acid-related GERD. This study suggests that persistent symptoms during vonoprazan therapy may be related to nonacid reflux, reflux hypersensitivity, functional heartburn, or esophageal motility disorders [27]. Consistent with this, another study comparing vonoprazan refractory vs. PPI refractory heartburn demonstrated that acid exposure time was normalized in the patients on vonoprazan, suggesting that heartburn refractory to vonoprazan is often not driven by persistent acid reflux [28]. Taken together, these findings suggest that vonoprazan, like classic PPIs, may be associated with persistent reflux-like symptoms in refractory patients despite effective acid suppression, possibly through mechanisms involving nonacid reflux or altered esophageal motility. In line with these clinical observations, our in vitro findings generate the hypothesis that vonoprazan-induced LES relaxation might theoretically contribute to persistent reflux symptoms. However, whether these direct tissue effects overcome intact neurohumoral compensatory mechanisms in vivo remains to be elucidated.

Next, we investigated whether vonoprazan modifies agonist-induced contractility after one hour of incubation. In isolated rat ileum and LES, vonoprazan inhibited carbachol- and serotonin-induced contractions in a concentration-dependent manner, with statistically significant reduction at concentrations ranging from 10 to 100 µM. Because carbachol induces gastrointestinal smooth muscle contraction through muscarinic receptor activation, the inhibition of carbachol-induced contractions suggests that vonoprazan functionally attenuates muscarinic contractile responses in isolated rat ileum and LES [29]. This observation is important because acetylcholine is considered a major excitatory neurotransmitter in all types of gastrointestinal smooth muscle [30]. Further supporting the muscarinic component of this interpretation, vonoprazan was classified as ‘ABS: 2’ on an IC_50_- and Cmax-based anticholinergic burden scale using a rat brain muscarinic radioligand binding assay, suggesting moderate muscarinic receptor-binding activity at usual dosing [31]. However, the experiments in the present study only demonstrate that contractions induced by carbachol are attenuated after vonoprazan incubation. Therefore, we cannot conclude that this effect is due to receptor antagonism.

Similarly, the attenuation of serotonin-induced contractions suggests that vonoprazan may also interfere with serotonergic contractile responses. This observation is noteworthy because serotonin is an important regulator of gastrointestinal motility, and a previous isolated rat ileum study has shown that serotonin can evoke contraction through a direct myogenic component that is not abolished by tetrodotoxin or atropine [32]. Therefore, the inhibitory effect of vonoprazan on serotonin-induced contractions suggests that its action is not restricted to cholinergic stimulation alone.

Vonoprazan attenuated contractions induced by both carbachol and serotonin in isolated rat ileum. However, these findings should not be interpreted as evidence of direct muscarinic or serotonergic receptor antagonism. The reduced contractile responses may instead reflect an effect on signaling mechanisms shared by these pathways, such as calcium entry, intracellular calcium mobilization, myosin light-chain phosphorylation, potassium channel activity, or excitation contraction coupling. Therefore, the present findings indicate a functional attenuation of carbachol- and serotonin-induced contractions rather than direct inhibition of their respective receptors.

Among these possible downstream mechanisms, altered Ca^2+^ entry may represent one potential explanation. This hypothesis is indirectly supported by isolated organ bath studies showing that classical PPIs can relax smooth muscle. For example, in isolated human myometrium tissues, pantoprazole was reported to reduce contractions induced by Ca^2+^ and high K^+^ [21]. Similarly, omeprazole decreased both spontaneous and Ca^2+^-induced contractions in isolated human myometrial smooth muscle [33]. However, this mechanism was not directly examined in the present study and should therefore be considered conjectural.

However, this study has several limitations. First, direct muscarinic or serotonergic receptor antagonism was not examined using receptor binding and signal transduction experiments. Therefore, the attenuation of carbachol- and serotonin-induced contractions cannot be attributed to direct blockade of their respective receptors. Second, the proposed mechanism other than functional suppression of muscarinic and serotonergic contractile responses involving changes in Ca^2+^ entry was not directly tested in the present study. Moreover, the available evidence from classical PPIs cannot be directly extrapolated to vonoprazan, which is a P-CAB. Therefore, this mechanistic interpretation should be considered hypothesis-generating and requires confirmation in future studies.

Second, the effects observed in the present study were obtained in isolated tissue preparations and may not directly reflect in vivo conditions. Moreover, caution must be exercised when extrapolating these in vitro findings to clinical outcomes, given the considerably high concentrations required for vonoprazan to exert its effects. The micromolar concentrations used in this study (1–100 µM) were specifically targeted because previous isolated tissue studies demonstrated that classical PPIs only begin to exert significant smooth muscle effects within this concentration range [18]. The concentrations required to produce these effects were in the high micromolar range and likely exceed clinically relevant free plasma levels achieved with standard dosing; therefore, caution is warranted when extrapolating these findings to systemic clinical effects. However, while systemic plasma exposure may not reach these levels, vonoprazan may achieve substantially higher local concentrations within the gastrointestinal lumen. In a pharmacokinetic study, PBPK/PD simulations predict stomach concentrations for vonoprazan ~1000-fold above plasma at 24 h in humans, making locally relevant high concentrations biologically plausible [34].

In conclusion, the current study demonstrates that vonoprazan has direct inhibitory effects on gastrointestinal smooth muscle, producing concentration-dependent relaxation in isolated rat ileum and LES, reducing the amplitude of spontaneous ileal contractions, and attenuating both carbachol- and serotonin-induced contractile responses after incubation. These findings demonstrate that vonoprazan can directly modulate gastrointestinal smooth muscle function independently of its acid suppressive action and provide a basis for the hypothesis that vonoprazan may influence clinical outcomes such as altered LES barrier function, delayed gastric emptying, constipation, or persistent reflux symptoms. Further clinical and translational studies are needed to clarify the clinical relevance and potential therapeutic implications of these effects.

## 4. Materials and Methods

### 4.1. Animals

A total number of 18 adult male Wistar albino rats, aged 6 to 8 weeks and weighing 200–250 g were obtained from the Experimental Medicine Research and Application Center of Selcuk University (Konya, Turkey). Animals were housed under standard laboratory conditions, including a controlled temperature of 22 ± 2 °C, relative humidity of 50–60%, and a 12 h light/dark cycle, with free access to standard chow and water. General anesthesia was induced with ketamine/xylazine. While under anesthesia, the lower esophageal sphincter and ileum tissues were excised.

### 4.2. Drugs and Solutions

Vonoprazan fumarate was obtained from MedChemExpress (Monmouth Junction, NJ, USA), carbamylcholine chloride from Sigma-Aldrich (St. Louis, MO, USA), serotonin hydrochloride from Alfa Aesar (Karlsruhe, Germany), and dimethyl sulfoxide (DMSO) from Merck (Darmstadt, Germany). The final concentration of vehicles in the organ bath was less than 0.1% *v*/*v*. DMSO at its respective final bath concentration had no effect on tissue contractility. All concentrations were expressed in molar (M) units. The composition of the Krebs–Henseleit solution (KHS) was as follows: NaCl, 135 mM; KCl, 5 mM; CaCl_2_, 2.5 mM; KH_2_PO_4_, 1.2 mM; MgSO_4_, 1.3 mM; NaHCO_3_, 20 mM; and glucose, 10 mM.

### 4.3. LES and Ileum Preparations

Esophagus, stomach, and ileum tissues were separated from surrounding tissues. The stomach was opened along the greater curvature, extending into the distal esophagus, and the gastroesophageal junction was exposed. The LES was identified as the thickened muscular region between the esophagus and stomach. The mucosal layer overlying the LES was carefully removed, and a transverse strip approximately 10 mm long and 2 mm wide was cut from this region [35]. Then, 10–20 mm segments were isolated from the ileum. Mucosa was retained in the ileal segments. One LES strip and one ileal tissue preparation were obtained from each rat and used in the experiments. Tissues were suspended under 1 g resting tension in 10 mL organ baths containing KHS maintained at 37 °C and continuously aerated with 95% O_2_ and 5% CO_2_. The preparations were allowed to equilibrate for 60 min, during which they were washed with fresh KHS at 15 min intervals [11].

### 4.4. Experimental Protocol

After equilibration, tissue preparations were randomly allocated to experimental protocols. For each experiment, tissues were initially contracted with 80 mM high-K^+^ KHS to assess tissue viability. Preparations showing prompt, measurable, and sustained contraction that returned to a stable baseline after washout were included in the analysis. One tissue preparation was excluded for failing the viability assessment.

First, direct effects of vonoprazan on isolated tissues were evaluated. Following precontraction with carbachol (1 µM), cumulative vonoprazan concentration response curves were obtained.

Second, the effect of vonoprazan on spontaneous contractions was evaluated. Spontaneous contractions during 5 min periods before and after the application of vonoprazan at different concentrations (1, 10, 100 µM) were compared. For spontaneous contraction experiments, post-treatment amplitude and frequency were normalized to the pretreatment baseline of the same ileum segment; therefore, each preparation served as its own baseline control.

Third, the effects of a 1 h incubation with vonoprazan at three different concentrations (1, 10, 100 µM) on contractions induced by cumulative carbachol (10^−8^–10^−4^ M) and serotonin (10^−8^–10^−4^ M) were investigated. For this purpose, in separate groups, baseline concentration–response curves to cumulative carbachol and serotonin were first obtained; after 1 h incubation with vonoprazan at each concentration, these responses were reassessed. Thus, each tissue preparation served as its own control.

### 4.5. Statistical Analysis

GraphPad Prism version 6.01 for Windows (San Diego, CA, USA) was used to conduct statistical analyses. Data distribution was assessed using the Shapiro–Wilk normality test, and homogeneity of variances was evaluated using Levene’s test. Normalized spontaneous contraction amplitude and frequency, together with pretreatment baseline values, were compared among groups using one-way ANOVA followed by Tukey’s post-hoc test. Direct vonoprazan relaxation concentration response curves were analyzed by nonlinear regression using a three-parameter log(agonist)-versus-response model, and −logEC_50_ values were compared using the extra-sum-of-squares F test. Agonist-induced concentration response curves were compared by repeated measures two-way ANOVA followed by Dunnett’s post-hoc test. The threshold for statistical significance was considered *p* < 0.05. Agonist-induced contractions were expressed as a percentage of the 80 mM high-K^+^ KHS-induced contractions. Data are presented as means ± standard error of the mean (SEM). The n values refer to the number of tissue preparations.

## Data Availability

The data presented in this study are available on request from the corresponding author.

## Supplementary Materials

The following supporting information can be downloaded at: https://www.mdpi.com/article/10.3390/ph19081199/s1, Figure S1: Pretreatment baseline spontaneous contraction amplitude and frequency in isolated rat ileum preparations; Figure S2: Representative original tracings of cumulative carbachol-induced contractions in isolated rat ileum; Figure S3: Representative original tracings of cumulative serotonin-induced contractions in isolated rat ileum preparations; Figure S4: Representative original tracings of cumulative carbachol-induced contractions in isolated rat lower esophageal sphincter preparations; Figure S5: Representative original tracings of cumulative serotonin-induced contractions in isolated rat lower esophageal sphincter preparations; Table S1: *p*-values for comparisons of carbachol induced contractions between control and vonoprazan treated groups at each carbachol concentration in rat ileum; Table S2: *p*-values for comparisons of serotonin induced contractions between control and vonoprazan treated groups at each serotonin concentration in rat ileum; Table S3: *p*-values for comparisons of carbachol induced contractions between control and vonoprazan treated groups at each carbachol concentration in rat LES; Table S4: *p*-values for comparisons of serotonin induced contractions between control and vonoprazan treated groups at each serotonin concentration in rat LES.
