# Supplementary material for: Effects of Vonoprazan on Isolated Rat Lower Esophageal Sphincter and Ileum
Source: Pharmaceuticals (Basel). 2026 Jul 31;19(8):1199. doi: 10.3390/ph19081199 (PMC13516295; doi:10.3390/ph19081199)
Supplement: Supplementary file 1 [file pharmaceuticals-19-01199-s001.zip › pharmaceuticals-4436133-supplementary.pdf]

Supplementary Table S1. *P*-values for comparisons of carbachol induced contractions between control and vonoprazan treated groups at each carbachol concentration in rat ileum (Figure 3A).

**log[Carbachol]**	**Control vs. vonoprazan 100 µM**	**Control vs. vonoprazan 10 µM**	**Control vs. vonoprazan 1 µM**
−8.0	0.9958	0.9962	>0.9999
−7.5	0.9885	0.9940	>0.9999
−7.0	0.8467	0.9402	0.9862
−6.5	0.0161	0.0554	0.7592
−6.0	<0.0001	<0.0001	0.7209
−5.5	<0.0001	<0.0001	0.4639
−5.0	<0.0001	0.1828	0.3993
−4.5	<0.0001	0.1771	0.1976
−4.0	<0.0001	0.1013	0.1845

Supplementary Table S2. *P*-values for comparisons of serotonin induced contractions between control and vonoprazan treated groups at each serotonin concentration in rat ileum (Figure 3B).

**log[Serotonin]**	**Control vs. vonoprazan 100 µM**	**Control vs. vonoprazan 10 µM**	**Control vs. vonoprazan 1 µM**
−8.0	0.9977	0.9984	0.9980
−7.5	0.9940	0.9849	0.9896
−7.0	0.9448	0.8739	>0.9999
−6.5	0.4317	0.4307	0.9946
−6.0	<0.0001	<0.0001	0.5276
−5.5	<0.0001	<0.0001	0.8011
−5.0	<0.0001	0.0003	0.9694
−4.5	<0.0001	0.0440	0.2415
−4.0	0.0095	0.7168	0.2170

Supplementary Table S3. *P*-values for comparisons of carbachol induced contractions between control and vonoprazan treated groups at each carbachol concentration in rat LES (Figure 4A).

log[Carbachol]	Control vs. vonoprazan 100 μM	Control vs. vonoprazan 10 μM	Control vs. vonoprazan 1 μM
−8.0	>0.9999	>0.9999	>0.9999
−7.5	0.9996	0.9996	0.9999
−7.0	0.9923	0.9953	0.9911
−6.5	0.9029	0.9428	0.9363
−6.0	0.0288	0.1127	0.8106
−5.5	0.0002	0.0230	0.8232
−5.0	<0.0001	0.2042	0.4700
−4.5	<0.0001	0.2250	0.2425
−4.0	<0.0001	0.6360	0.1430

Supplementary Table S4. *P*-values for comparisons of serotonin induced contractions between control and vonoprazan treated groups at each serotonin concentration in rat LES (Figure 4B).

log[Serotonin]	Control vs. vonoprazan 100 μM	Control vs. vonoprazan 10 μM	Control vs. vonoprazan 1 μM
−8.0	0.9997	>0.9999	>0.9999
−7.5	0.9999	>0.9999	0.9997
−7.0	0.9767	0.9979	>0.9999
−6.5	0.6733	0.7353	0.9373
−6.0	0.3811	0.5173	>0.9999
−5.5	0.0021	0.0156	>0.9999
−5.0	<0.0001	0.0015	0.9452
−4.5	<0.0001	0.0021	0.8956
−4.0	0.0002	0.4308	0.6413

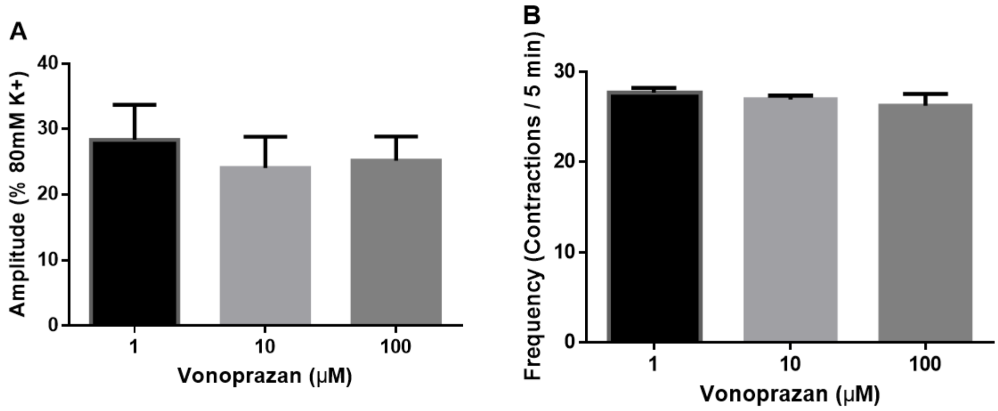

Supplementary Figure S1. Pretreatment baseline spontaneous contraction amplitude (A) and frequency (B) in isolated rat ileum preparations assigned to subsequent incubation with vonoprazan at 1, 10, or 100 µM. Amplitude was expressed as a percentage of the response to 80 mM K^+, and frequency was expressed as the number of contractions per 5 min. No significant differences were observed among the groups ($p > 0.05$). Data are presented as mean ± SEM.

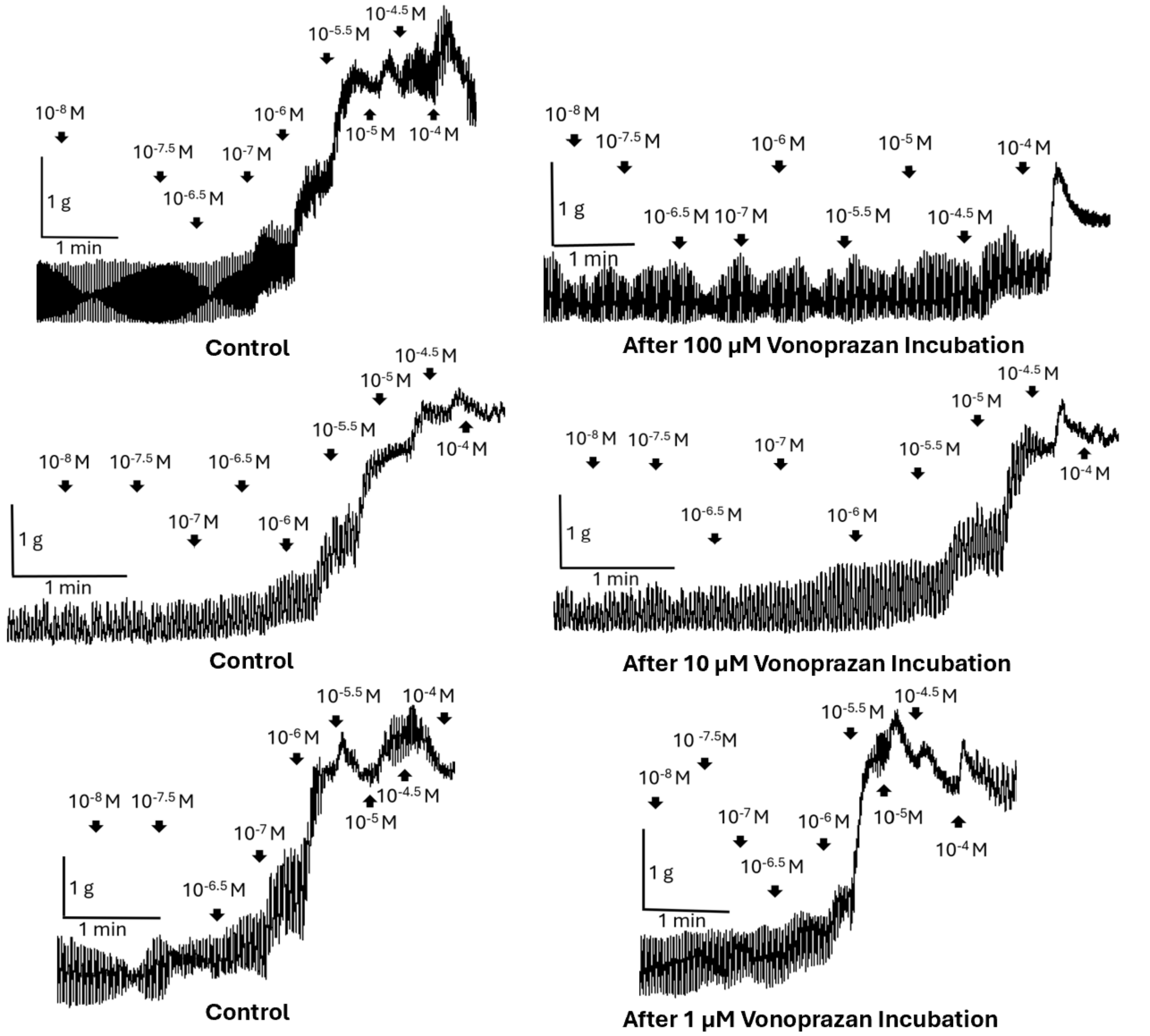

Supplementary Figure S2. Representative original tracings of cumulative carbachol-induced contractions in isolated rat ileum under control conditions and after 1 h incubation with vonoprazan at 100 µM (top), 10 µM (middle), or 1 µM (bottom). Arrows indicate the cumulative addition of carbachol at concentrations ranging from 10^{-8} to 10^{-4} M in 0.5-log increments. Calibration bars represent 1 g of tension and 1 min. These recordings correspond to the concentration response data presented in Figure 3A.

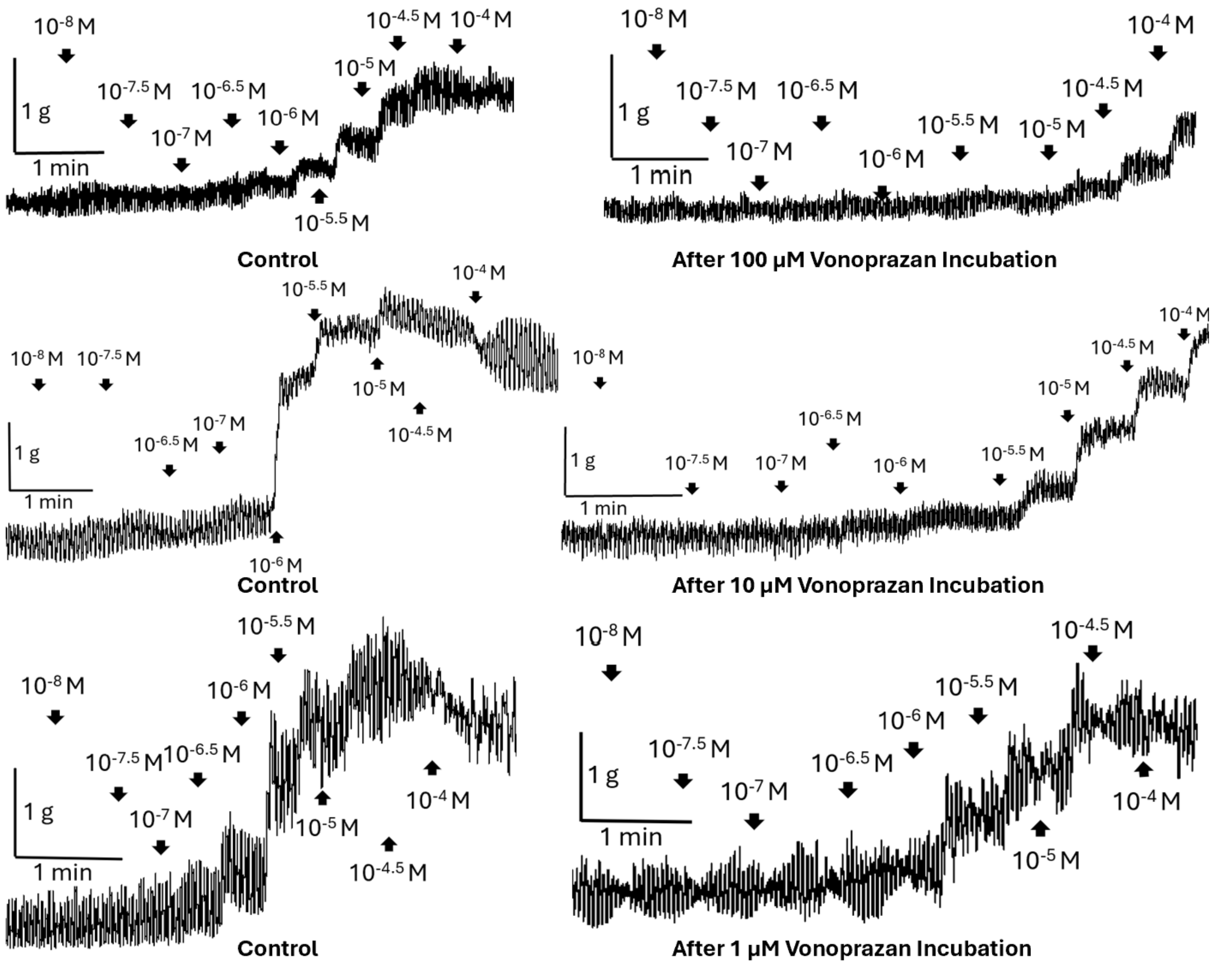

Supplementary Figure S3. Representative original tracings of cumulative serotonin-induced contractions in isolated rat ileum preparations under control conditions and after 1 h incubation with vonoprazan at 100 µM (top), 10 µM (middle), or 1 µM (bottom). Arrows indicate the cumulative addition of serotonin at concentrations ranging from 10^{-8} to 10^{-4} M in 0.5-log increments. Calibration bars represent 1 g of tension and 1 min. These recordings correspond to the concentration response data presented in Figure 3B.

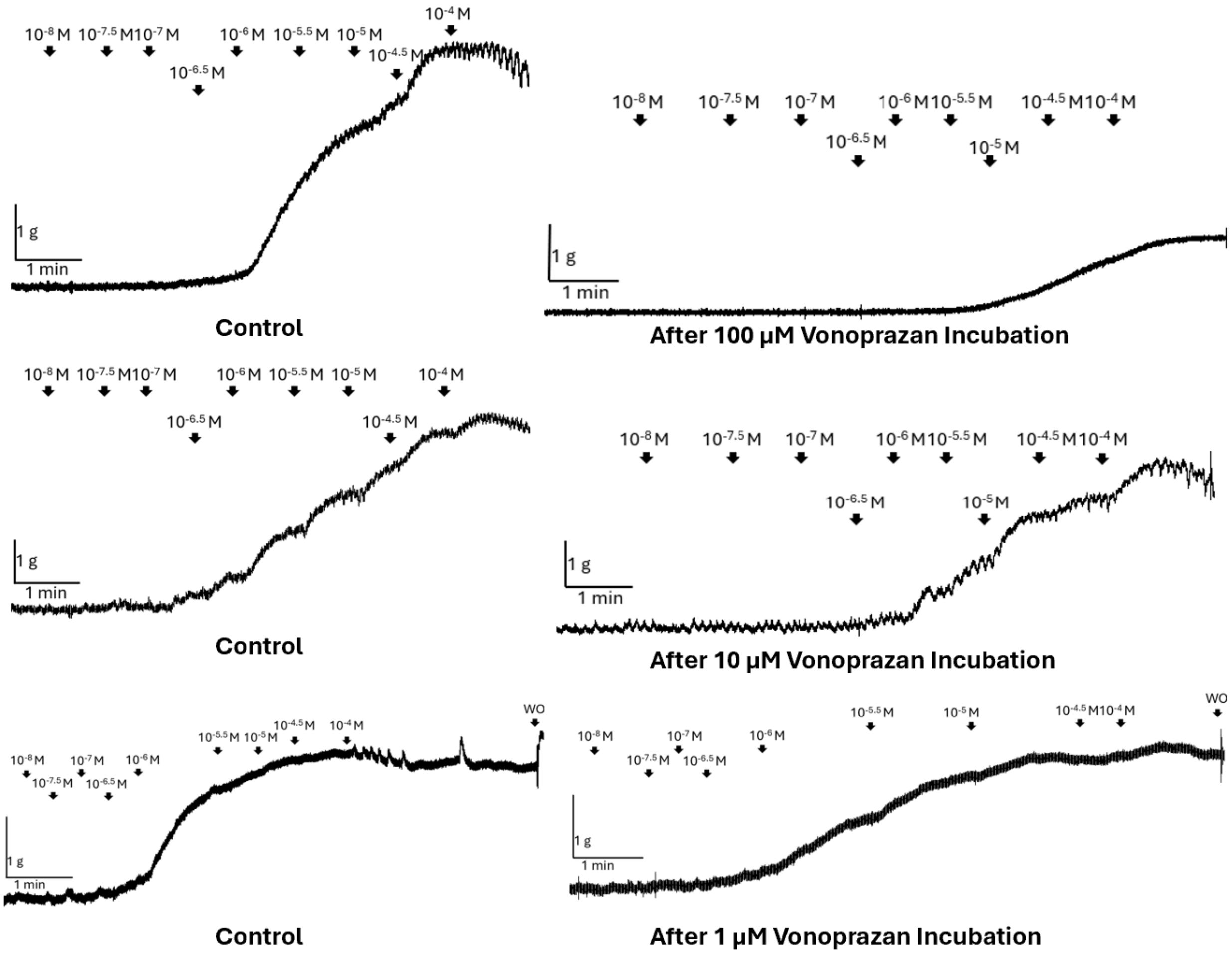

Supplementary Figure S4. Representative original tracings of cumulative carbachol-induced contractions in isolated rat lower esophageal sphincter preparations under control conditions and after 1 h incubation with vonoprazan at 100 µM (top), 10 µM (middle), or 1 µM (bottom). Arrows indicate the cumulative addition of carbachol at concentrations ranging from 10^{-8} to 10^{-4} M in 0.5-log increments. Calibration bars represent 1 g of tension and 1 min. WO indicates washout. These recordings correspond to the concentration response data presented in Figure 4A.

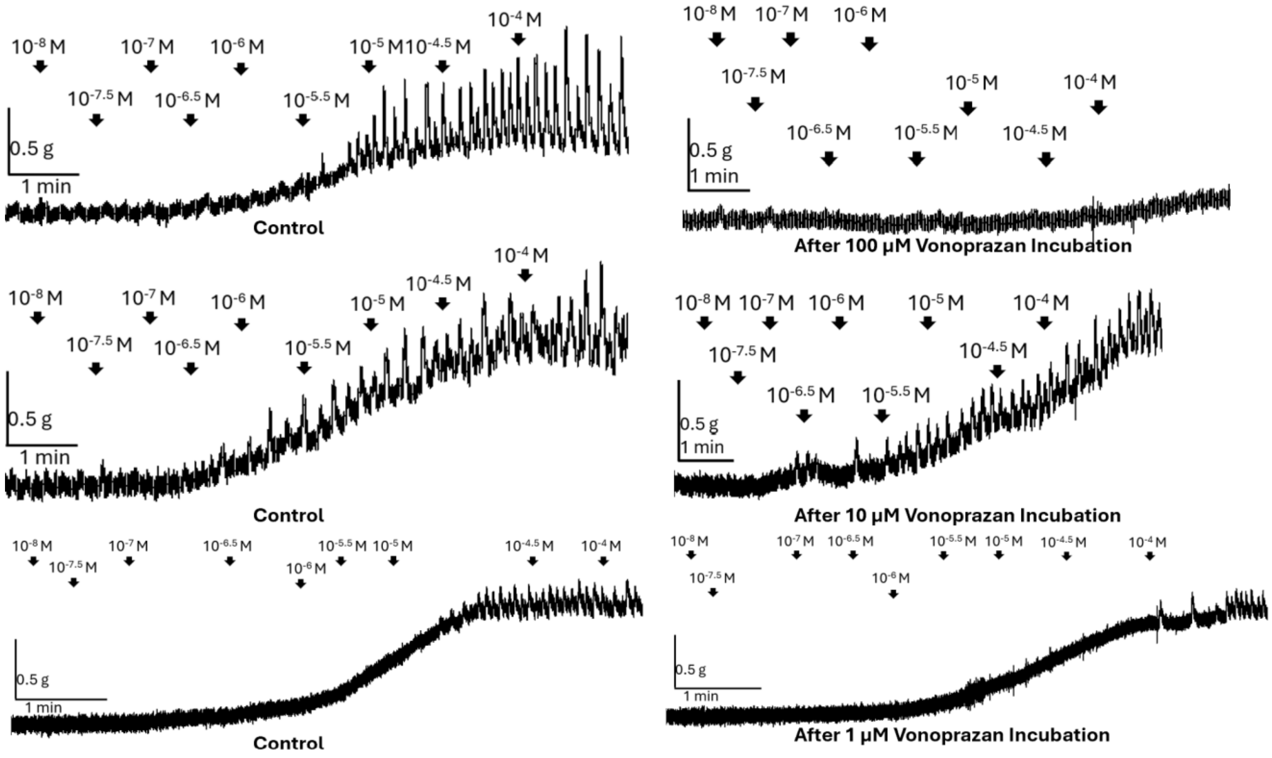

Supplementary Figure S5. Representative original tracings of cumulative serotonin-induced contractions in isolated rat lower esophageal sphincter preparations under control conditions and after 1 h incubation with vonoprazan at 100 μM (top), 10 μM (middle), or 1 μM (bottom). Arrows indicate the cumulative addition of serotonin at concentrations ranging from 10^{-8} to 10^{-4} M in 0.5-log increments. Calibration bars represent 0.5 g of tension and 1 min. These recordings correspond to the concentration response data presented in Figure 4.
